# Evaluation of Helicobacter pylori and Small Intestinal Bacterial Overgrowth in Subjects With Rosacea

**DOI:** 10.7759/cureus.72363

**Published:** 2024-10-25

**Authors:** Jessie M Nelson, Jason M Rizzo, Rachel K Greene, Kathryn Fahlstrom, Jonathan P Troost, Yolanda R Helfrich, Mio Nakamura

**Affiliations:** 1 Dermatology, University of Michigan, Ann Arbor, USA; 2 Dermatology, The Woodruff Institute for Dermatology, Bonita Springs, USA; 3 Biostatistics, Michigan Institute for Clinical and Health Research, University of Michigan, Ann Arbor, USA

**Keywords:** h. pylori, immunity, inflammation, rosacea, sibo

## Abstract

Background: Systemic abnormalities in the immune system may contribute to rosacea pathogenesis. Several studies have found a higher prevalence of abnormal bacterial growth, such as *Helicobacter pylori *(*H. pylori*) and small intestinal bacterial overgrowth (SIBO) in rosacea subjects. However, discrepancies remain in the literature, likely perpetuated by inconsistent testing methods and incomplete controlling for potential confounders.

Objective: We aimed to evaluate the prevalence of *H. pylori* and SIBO in rosacea subjects after controlling for several potential confounders.

Methods: This cross-sectional study evaluated subjects with papulopustular or erythematotelangiectatic rosacea. Subjects with previous or existing gastrointestinal (GI) disease, GI surgery, autoimmune disorders, immunosuppression, or significant comorbidities were excluded. Certain medication use (antibiotics, steroids, GI-modulating medications, anti-inflammatories) required an appropriate washout period. Rosacea history and severity were assessed. Subjects answered questions regarding their rosacea and GI health. *H. pylori *andSIBO were evaluated by ^13^C-urea breath test and glucose-breath test methods, respectively.

Results: Of 27 subjects, 14.8% (N=4) tested positive for *H. pylori* and 33.3% (N=9) tested positive for SIBO. Compared to the general population prevalence, the proportion of* H. pylori* in the rosacea cohort was significantly less (p=0.02). Though the estimated population prevalence of SIBO had a wider range, compared to midrange, the prevalence of SIBO in the rosacea cohort was greater (p<0.001). There were no significant associations between demographics, rosacea characteristics, or GI symptoms and *H. pylori* or SIBO positivity.

Conclusion: When eliminating several potential confounders, SIBO is more prevalent in subjects with rosacea compared to the general population. Thus, SIBO may be associated with rosacea, though it remains incompletely understood whether SIBO itself contributes to rosacea pathophysiology or rather SIBO prevalence and rosacea are both downstream effects of abnormalities in systemic immunity. Future studies are warranted to elucidate this relationship further, though this observed association may be promising for novel therapeutic targets in rosacea treatment.

## Introduction

Rosacea is a chronic inflammatory skin condition affecting more than five percent of the general population [1]. Primarily involving the central face, it often presents with episodic flushing, non-transient erythema, papules, pustules, and telangiectasia. There are four primary subtypes of rosacea based on cutaneous clinical features: erythematotelangiectatic (ETR), papulopustular (PPR), phymatous, and ocular. Flushing can be triggered by various environmental stimuli such as sunlight, heat, alcohol, or spicy food and may be associated with burning or stinging sensations. Onset is typically between 30 and 50 years of age and is more commonly diagnosed in individuals with fair skin phototypes [1]. Though generally regarded to have a female predominance, some recent reports suggest that there may be no specific sex predilection [2].

Rosacea has also been shown to be associated with various systemic conditions, including gastrointestinal (GI), neurologic, psychiatric, cardiovascular, metabolic, and autoimmune diseases [3]. Despite several theories that propose dysregulation of immunity, generation of reactive oxygen species, aberrant neurovascular signaling, and microorganismal overgrowth, the exact pathogenesis of rosacea remains incompletely understood [4]. 

Recently, the role of the gut microbiome in rosacea has become the focus of attention. With GI conditions being the most frequently reported comorbidity, several studies have investigated extracutaneous bacterial overgrowth in the GI tract as a biomarker for systemic abnormalities in immunity. Many reports have identified a greater proportion of *Helicobacter pylori *(*H. pylori*) and small intestinal bacterial overgrowth (SIBO) in rosacea subjects [5,6]. However, the consistency of these findings remains highly variable in the literature, perhaps due to inconsistent testing methods and incomplete controlling for potential confounders [7,8].

To address such limitations and contribute to the available literature, we tested a cohort of rosacea subjects for *H. pylori *and SIBO while controlling for potential confounding variables such as rosacea subtype, GI disease, prior GI surgery, antibiotics, non-steroidal anti-inflammatory drugs (NSAIDs), GI-modulating medications, immunosuppression, autoimmune disease, or significant comorbidities. We aimed to better ascertain the prevalence of *H. pylori* and SIBO in rosacea subjects, identify potential associations with clinical characteristics, and supplement the discussion of the relationship between rosacea and bacterial overgrowth as it relates to abnormalities in immunity.

## Materials and methods

This cross-sectional study was approved by the Michigan Medicine Institutional Review Board. Subjects were recruited directly from the institution’s dermatology clinics or through a recruitment website and were enrolled between 2018 and 2022.

Inclusion and exclusion criteria

Eligible subjects were required to be at least 18 years old and have a clinical diagnosis of rosacea (either ETR or PPR subtypes). Isolated phymatous or ocular subtypes were excluded, but those with concomitant phymatous or ocular features in addition to their primary ETR or PPR subtype were permitted. Pregnant or breastfeeding individuals were excluded. Additional exclusion criteria were used to eliminate various potential confounders in evaluating rosacea and GI bacterial colonization and to ensure subject safety over the study period. Therefore, subjects were excluded if they had a history of GI surgery, colonic purging or bowel preparation within two months, prior or current GI disease (celiac disease, inflammatory bowel disease, irritable bowel syndrome, lipase deficiency, pancreatic insufficiency, cystic fibrosis), renal disease, liver disease, immunosuppression, autoimmune disorder, non-steroidal anti-inflammatory medication use greater than twice weekly, or other significant medical history that may interfere with study procedures or accuracy as determined by the investigators.

Subjects who were taking certain medications were permitted to participate in the study following completion of a pre-specified washout period: antacids (24 hours), histamine antagonists (three days), proton pump inhibitors (seven days), bismuths, probiotics, topical or oral antibiotics, topical or systemic corticosteroids, topical or systemic acne treatments (30 days), laxatives, opiates, prokinetics (eight weeks), and systemic retinoids (12 weeks).

Study procedures

Written informed consent was obtained. The subject’s pertinent medical history was reviewed, and a skin exam was performed to evaluate the type and extent of rosacea. Eligible subjects were then counseled to adhere to standard dietary practices prior to *H. pylori *and SIBO breath testing to ensure accuracy of breath test results: low carbohydrate diet for 48 hours, nothing by mouth for eight hours, no smoking or chewing gum for eight hours, and no physical exercise for eight hours.

Subjects were then administered a clinical assessment questionnaire to collect information on known flushing triggers, associated burning or stinging, skin irritants, and response to prior rosacea treatments. Subjects also completed a modified patient assessment of constipation-symptoms (PAC-SYM) questionnaire [9]. A thorough evaluation of the subject’s skin was performed, including completion of the verified rosacea clinical scorecard, a global assessment of rosacea features, classification into a primary rosacea type (ETR or PPR), and Fitzpatrick skin phototyping [10]. Breath tests for *H. pylori* and SIBO were performed by a trained technician.

H. pylori Breath Testing

*H. pylori *colonization positivity was determined by ^13^C-labeled urea breath testing (^13^C-UBT). ^13^C-labeled urea is hydrolyzed to NH_3_ and CO_2_ by *H. pylori* in the upper GI tract. Subjects were exhaled into a collection bag to obtain a baseline value of ^13^CO_2_ using a single gas isotope ratio mass spectrometer. They then ingested a solution of 100 mg of ^13^C-labeled urea and 1.4 g citric acid over one minute. The citric acid aims to reduce contamination by urease-producing mouth flora [11]. A second breath sample was obtained 15 minutes after ingestion of the solution. A positive test was concluded if the difference between the baseline and 15-minute sample exceeded five parts per 1000 of ^13^CO_2_. 

SIBO Breath Testing

Evaluation for SIBO was performed by glucose breath testing, which is supported as the best, non-invasive modality for SIBO diagnosis [12]. In SIBO, the bacteria present in the proximal aspect of the small intestine ferment glucose before it can be absorbed by the colon, resulting in increased breath excretion of hydrogen and/or methane above baseline values. In accordance with the Rome Consensus Conference protocol, the subjects ingested a solution of 50 g glucose with 120 mL water over five minutes after a baseline breath sample was obtained. Then, breath samples were evaluated every 15 minutes for a total of 120 minutes. The first 100 cc of each breath sample was discarded, and only the last 50 cc was evaluated by gas chromatography. A positive result was defined by an increase in hydrogen or methane by at least 12 ppm from baseline that is sustained for greater than or equal to two consecutive readings [13].

Statistics

All data were analyzed by a biostatistician (author JPT) using Statistical Analysis Software (SAS v9.4, IBM Corp., Armonk, New York, USA). Binary predictors were assessed using Chi-square tests. Ordinal and continuous variables were both assessed using the Kruskal-Wallis test. P<0.05 was considered statistically significant.

## Results

Subject demographics and clinical characteristics

A total of 30 subjects were recruited; 27 completed all study procedures. Most subjects were female (70%), with a mean age of 48.3 years (range 25-70). About 81% of subjects had ETR (n=22), whereas 19% had PPR (n=5). Most subjects had mild or moderate rosacea based on a global assessment of rosacea severity (48% and 41%, respectively). A Fitzpatrick Skin Phototype score of 2 was the most common (56%), followed by 3 (26%) and 1 (11%).

*H. pylori *and SIBO

Four subjects (14.8%) tested positive for *H. pylori.* Nine subjects (33.3%) tested positive for SIBO. Only one of the 13 positive subjects (7.7%) tested positive for both *H. pylori *and SIBO. To evaluate significance, sample proportions were compared to reported population prevalence for each.

It is estimated that the general United States population prevalence of *H. pylori* colonization is between 0.35 and 0.4 [14]. In our rosacea sample population, significantly fewer subjects were colonized with *H. pylori* as compared to the numbers reported in the general population (0.15 sample vs 0.38 mid-range population, p=0.02). A comparison with the upper range prevalence of 0.4 also yielded a significant difference (p=0.03).

Estimates regarding SIBO prevalence in the general population vary more widely as compared to estimates for *H. pylori *prevalence. This is partly due to SIBO being assessed less frequently than *H. pylori* colonization. Additionally, detection methods used to detect SIBO and their accuracies vary considerably [15]. From our review, most reports estimate a general population prevalence of 0.025 to 0.22 [16]. In our rosacea sample population, significantly more subjects were colonized with SIBO as compared to the mid-estimation in the general population (0.33 sample vs 0.12 mid-range population, p<0.001). However, a comparison to the 0.22 upper-limit prevalence estimation demonstrated no such significance (p=0.16).

Associations between subject characteristics and *H. pylori *and SIBO

Numerous rosacea features, demographics, and GI symptoms were assessed for associations with *H. pylori *and SIBO positivity. A summary of the results can be found in Tables 1-3. There were no significant associations between rosacea features and demographic information and *H. pylori *or SIBO positivity (Table 1).

**Table 1:** H. pylori and SIBO breath test results by clinical characteristics and rosacea scorecard. ^a^Binary predictors were assessed using Chi-square tests. ^b^Ordinal and continuous variables were both assessed using the Kruskal-Wallis test. P<0.05 was considered statistically significant. Numerical variables are reported as mean (standard deviation), categorical variables are reported as N (%). *H. pylori*: *Helicobacter pylori*, SIBO: small intestinal bacterial overgrowth.

Clinical characteristics	Overall	H. pylori or SIBO			H. pylori			SIBO		
		Positive	Negative	p-value	Positive	Negative	p-value	Positive	Negative	p-value
Sex										
Female	19 (70)	9 (75)	10 (67)	0.64^a^	4 (100)	15 (65)	0.16^a^	6 (67)	13 (72)	0.77^a^
Age										
Years	48.3 (14.9)	49.1 (13.3)	47.7 (16.4)	0.94^b^	50.8 (14.4)	47.9 (15.2)	0.68^b^	46.9 (13.5)	49.1 (15.8)	0.62^b^
Fitzpatrick skin phototype										
1	3 (11)	2 (17)	1 (7)	0.39^b^	1 (25)	2 (9)	0.11^b^	2 (22)	1 (6)	0.69^b^
2	15 (56)	7 (58)	8 (53)		3 (75)	12 (52)		4 (44)	11 (61)	
3	7 (26)	2 (17)	5 (33)		0 (0)	7 (30)		2 (22)	5 (28)	
4	1 (4)	0 (0)	1 (7)		0 (0)	1 (4)		0 (0)	1 (6)	
5	1 (4)	1 (8)	0 (0)		0 (0)	1 (4)		1 (11)	0 (0)	
Primary rosacea type										
ETR	22 (81)	11 (92)	11 (73)	0.22^a^	4 (100)	18 (78)	0.30^a^	8 (89)	14 (78)	0.48^a^
PPR	5 (19)	1 (8)	4 (27)		0 (0)	5 (22)		1 (11)	4 (22)	
Rosacea severity										
Absent	3 (11)	2 (17)	1 (7)	0.39^b^	1 (25)	2 (9)	0.07^b ^	1 (11)	2 (11)	0.82^b^
Mild	13 (48)	6 (50)	7 (47)		3 (75)	10 (43)		4 (44)	9 (50)	
Moderate	11 (41)	4 (33)	7 (47)		0 (0)	11 (48)		4 (44)	7 (39)	
Rosacea scorecard (primary)										
Flushing										
Absent	7 (26)	4 (33)	3 (20)	0.29^b^	2 (50)	5 (22)	0.14^b^	2 (22)	5 (28)	1.00^b^
Mild	13 (48)	6 (50)	7 (47)		2 (50)	11 (48)		5 (56)	8 (44)	
Moderate	7 (26)	2 (17)	5 (33)		0 (0)	7 (30)		2 (22)	5 (28)	
Non-transient erythema										
Absent	2 (7)	1 (8)	1 (7)	0.68^b^	0 (0)	2 (9)	0.57^b^	1 (11)	1 (6)	0.57^b^
Mild	13 (48)	5 (42)	8 (53)		3 (75)	10 (43)		3 (33)	10 (56)	
Moderate	12 (44)	6 (50)	6 (40)		1 (25)	11 (48)		5 (56)	7 (39)	
Papules/pustules										
Absent	10 (37)	5 (42)	5 (33)	0.30^b^	1 (25)	9 (39)	0.85^b^	4 (44)	6 (33)	0.39^b^
Mild	11 (41)	6 (50)	5 (33)		3 (75)	8 (35)		4 (44)	7 (39)	
Moderate	6 (22)	1 (8)	5 (33)		0 (0)	6 (26)		1 (11)	5 (28)	
Telangiectasia										
Absent	6 (22)	3 (25)	3 (20)	0.81^b^	1 (25)	5 (22)	0.91^b^	3 (33)	3 (17)	0.68^b^
Mild	9 (33)	3 (25)	6 (40)		1 (25)	8 (35)		2 (22)	7 (39)	
Moderate	12 (44)	6 (50)	6 (40)		2 (50)	10 (43)		4 (44)	8 (44)	
Rosacea scorecard (secondary)										
Burning/stinging										
Absent	17 (63)	8 (67)	9 (60)	0.64^b^	2 (50)	15 (65)	0.63^b^	6 (67)	11 (61)	0.72^b^
Mild	9 (33)	4 (33)	5 (33)		2 (50)	7 (30)		3 (33)	6 (33)	
Moderate	1 (4)	0 (0)	1 (7)		0 (0)	1 (4)		0 (0)	1 (6)	
Plaques										
Absent	25 (93)	12 (100)	13 (87)	0.20^b^	4 (100)	21 (91)	0.55^b^	9 (100)	16 (89)	0.31^b^
Mild	2 (7)	0 (0)	2 (13)		0 (0)	2 (9)		0 (0)	2 (11)	
Dry appearance										
Absent	11 (41)	4 (33)	7 (47)	0.51^b^	1 (25)	10 (43)	0.30^b^	3 (33)	8 (44)	0.86^b^
Mild	14 (52)	7 (58)	7 (47)		2 (50)	12 (52)		6 (67)	8 (44)	
Moderate	2 (7)	1 (8)	1 (7)		1 (25)	1 (4)		0 (0)	2 (11)	
Edema										
Absent	22 (81)	10 (83)	12 (80)	0.83^b^	3 (75)	19 (83)	0.72^b^	8 (89)	14 (78)	0.49^b^
Mild	5 (19)	2 (17)	3 (20)		1 (25)	4 (17)		1 (11)	4 (22)	
Ocular manifestations										
Absent	14 (52)	6 (50)	8 (53)	0.87^b^	1 (25)	13 (57)	0.38^b^	5 (56)	9 (50)	0.62^b^
Mild	11 (41)	6 (50)	5 (33)		3 (75)	8 (35)		4 (44)	7 (39)	
Moderate	1 (4)	0 (0)	1 (7)		0 (0)	1 (4)		0 (0)	1 (6)	
Severe	1 (4)	0 (0)	1 (7)		0 (0)	1 (4)		0 (0)	1 (6)	
Peripheral location										
Absent	21 (78)	10 (83)	11 (73)	0.53^a^	3 (75)	18 (78)	0.88^a^	8 (89)	13 (72)	0.33^a^
Present	6 (22)	2 (17)	4 (27)		1 (25)	5 (22)		1 (11)	5 (28)	
Phymatous changes										
Absent	21 (78)	9 (75)	12 (80)	0.76^b^	3 (75)	18 (78)	0.89^b^	7 (78)	14 (78)	1.00^b^
Mild	6 (22)	3 (25)	3 (20)		1 (25)	5 (22)		2 (22)	4 (22)	

Likewise, there were no significant associations with prior treatment response and *H. pylori *or SIBO positivity (Table 2).

**Table 2:** H. pylori and SIBO breath test results by prior treatment responses. ^a^Ordinal were assessed using the Kruskal-Wallis test. P<0.05 was considered statistically significant. Categorical variables are reported as N (%). *H. pylori*: *Helicobacter pylori*, SIBO: small intestinal bacterial overgrowth.

Prior treatment response	Overall	H. pylori or SIBO			H. pylori			SIBO		
		Positive	Negative	p-value	Positive	Negative	p-value	Positive	Negative	p-value
Metronidazole cream										
None	7 (26)	3 (25)	4 (27)	0.94^a^	0 (0)	7 (30)	0.12^a^	3 (33)	4 (22)	0.29^a^
Poor	14 (52)	6 (50)	8 (53)		2 (50)	12 (52)		5 (56)	9 (50)	
Fair	2 (7)	2 (17)	0 (0)		1 (25)	1 (4)		1 (11)	1 (6)	
Good	4 (15)	1 (8)	3 (20)		1 (25)	3 (13)		0 (0)	4 (22)	
Azelaic acid										
None	18 (67)	8 (67)	10 (67)	1.00^a^	3 (75)	15 (65)	0.65^a^	6 (67)	12 (67)	0.95^a^
Poor	7 (26)	3 (25)	4 (27)		1 (25)	6 (26)		2 (22)	5 (28)	
Fair	1 (4)	1 (8)	0 (0)		0 (0)	1 (4)		1 (11)	0 (0)	
Good	1 (4)	0 (0)	1 (7)		0 (0)	1 (4)		0 (0)	1 (6)	
Oral tetracyclines										
None	12 (44)	6 (50)	6 (40)	0.61^a^	3 (75)	9 (39)	0.13^a^	3 (33)	9 (50)	0.50^a^
Poor	6 (22)	3 (25)	3 (20)		1 (25)	5 (22)		3 (33)	3 (17)	
Fair	1 (4)	0 (0)	1 (7)		0 (0)	1 (4)		0 (0)	1 (6)	
Good	4 (15)	1 (8)	3 (20)		0 (0)	4 (17)		1 (11)	3 (17)	
Excellent	4 (15)	2 (17)	2 (13)		0 (0)	4 (17)		2 (22)	2 (11)	
Sulfur-based topical										
None	22 (81)	11 (92)	11 (73)	0.22^a^	4 (100)	18 (78)	0.31^a^	8 (89)	14 (78)	0.47^a^
Poor	4 (15)	1 (8)	3 (20)		0 (0)	4 (17)		1 (11)	3 (17)	
Good	1 (4)	0 (0)	1 (7)		0 (0)	1 (4)		0 (0)	1 (6)	
Topical retinoids										
None	20 (74)	9 (75)	11 (73)	0.95^a^	3 (75)	17 (74)	0.89^a^	7 (78)	13 (72)	0.84^a^
Poor	5 (19)	2 (17)	3 (20)		1 (25)	4 (17)		1 (11)	4 (22)	
Fair	2 (7)	1 (8)	1 (7)		0 (0)	2 (9)		1 (11)	1 (6)	

When evaluating constipation symptoms, “minutes attempting to defecate” was significantly higher for SIBO-positive as compared to negative subjects (average 5.7 minutes vs. 3.7 minutes, P=0.03). Other variables like “abdominal pain” and “bloating” had low, but insignificant, p-values (P=0.06 and P=0.05, respectively) (Table 3).

**Table 3:** Constipation questionnaire by H. pylori and SIBO breath test results. ^a^Ordinal and continuous variables were both assessed using the Kruskal-Wallis test. P<0.05 was considered statistically significant.*Denotes significance at α=0.05. Numerical variables reported as mean (standard deviation), Categorical variables reported as N (%). *H. pylori*: *Helicobacter pylori*, SIBO: small intestinal bacterial overgrowth, BM: bowel movement.

Constipation symptoms	Overall	*H. pylori *or SIBO			H. pylori			SIBO		
		Positive	Negative	p-value	Positive	Negative	p-value	Positive	Negative	p-value
Abdominal discomfort										
Absent	16 (59)	6 (50)	10 (67)	0.49^a^	1 (25)	15 (65)	0.15^a^	5 (56)	11 (61)	0.98^a^
Mild	6 (22)	4 (33)	2 (13)		2 (50)	4 (17)		3 (33)	3 (17)	
Moderate	4 (15)	1 (8)	3 (20)		0 (0)	4 (17)		1 (11)	3 (17)	
Severe	1 (4)	1 (8)	0 (0)		1 (25)	0 (0)		0 (0)	1 (6)	
Abdominal pain										
Absent	22 (81)	9 (75)	13 (87)	0.41^a^	2 (50)	20 (87)	0.06^a^	8 (89)	14 (78)	0.47^a^
Mild	4 (15)	2 (17)	2 (13)		1 (25)	3 (13)		1 (11)	3 (17)	
Severe	1 (4)	1 (8)	0 (0)		1 (25)	0 (0)		0 (0)	1 (6)	
Bloating										
Absent	12 (44)	4 (33)	8 (53)	0.23^a^	0 (0)	12 (52)	0.05^a^	4 (44)	8 (44)	0.96^a^
Mild	8 (30)	4 (33)	4 (27)		2 (50)	6 (26)		3 (33)	5 (28)	
Moderate	5 (19)	2 (17)	3 (20)		1 (25)	4 (17)		1 (11)	4 (22)	
Severe	2 (7)	2 (17)	0 (0)		1 (25)	1 (4)		1 (11)	1 (6)	
Stomach cramps										
Absent	20 (74)	8 (67)	12 (80)	0.50^a^	2 (50)	18 (78)	0.23^a^	6 (67)	14 (78)	0.76^a^
Mild	2 (7)	2 (17)	0 (0)		1 (25)	1 (4)		2 (22)	0 (0)	
Moderate	4 (15)	1 (8)	3 (20)		0 (0)	4 (17)		1 (11)	3 (17)	
Severe	1 (4)	1 (8)	0 (0)		1 (25)	0 (0)		0 (0)	1 (6)	
Painful BMs										
Absent	24 (89)	10 (83)	14 (93)	0.45^a^	3 (75)	21 (91)	0.32^a^	8 (89)	16 (89)	0.92^a^
Mild	1 (4)	1 (8)	0 (0)		0 (0)	1 (4)		1 (11)	0 (0)	
Moderate	2 (7)	1 (8)	1 (7)		1 (25)	1 (4)		0 (0)	2 (11)	
Rectal burning with BMs										
Absent	22 (81)	10 (83)	12 (80)	0.94^a^	3 (75)	19 (83)	0.65^a^	8 (89)	14 (78)	0.54^a^
Mild	2 (7)	0 (0)	2 (13)		0 (0)	2 (9)		0 (0)	2 (11)	
Moderate	3 (11)	2 (17)	1 (7)		1 (25)	2 (9)		1 (11)	2 (11)	
Rectal bleeding/tearing with BMs										
Absent	25 (93)	12 (100)	12 (87)	0.20^a^	4 (100)	21 (91)	0.55^a^	9 (100)	16 (89)	0.31^a^
Mild	2 (7)	0 (0)	2 (13)		0 (0)	2 (9)		0 (0)	2 (11)	
Incomplete BMs										
Absent	17 (63)	6 (50)	11 (73)	0.36^a^	1 (25)	16 (70)	0.15^a^	6 (67)	11 (61)	0.68^a^
Mild	5 (19)	4 (33)	1 (7)		2 (50)	3 (13)		2 (22)	3 (17)	
Moderate	5 (19)	2 (17)	3 (20)		1 (25)	4 (17)		1 (11)	4 (22)	
Hard BMs										
Absent	20 (74)	10 (83)	10 (67)	0.28^a^	3 (75)	17 (74)	0.89^a^	8 (89)	12 (67)	0.20^a^
Mild	5 (19)	2 (17)	3 (20)		1 (25)	4 (17)		1 (11)	4 (22)	
Moderate	2 (7)	0 (0)	2 (13)		0 (0)	2 (9)		0 (0)	2 (11)	
Small BMs										
Absent	20 (74)	9 (75)	11 (73)	0.77^a^	2 (50)	18 (78)	0.33^a^	7 (78)	13 (72)	0.66^a^
Mild	5 (19)	3 (25)	2 (13)		2 (50)	3 (13)		2 (22)	3 (17)	
Moderate	2 (7)	0 (0)	2 (13)		0 (0)	2 (9)		0 (0)	2 (11)	
Straining with BMs										
Absent	14 (52)	6 (50)	8 (53)	0.89^a^	2 (50)	12 (52)	0.82^a^	5 (56)	9 (50)	0.73^a^
Mild	9 (33)	5 (42)	4 (27)		2 (50)	7 (30)		3 (33)	6 (33)	
Moderate	4 (15)	1 (8)	3 (20)		0 (0)	4 (17)		1 (11)	3 (17)	
Time attempting BM										
Minutes	4.3 (2.98)	5.2 (2.87)	3.7 (3.00)	0.08^a^	3.8 (2.02)	4.4 (3.12)	0.96^a^	5.7 (2.89)	3.7 (2.87)	0.03^a*^
Bristol stool type of last BM										
2	2 (7)	1 (8)	1 (7)	0.63^a^	0 (0)	2 (9)	0.39^a^	1 (11)	1 (6)	0.25^a^
3	6 (22)	3 (25)	3 (20)		0 (0)	6 (26)		3 (33)	3 (17)	
4	11 (41)	5 (42)	6 (40)		3 (75)	8 (35)		3 (33)	8 (44)	
5	3 (11)	1 (8)	2 (13)		0 (0)	3 (13)		1 (11)	2 (11)	
6	2 (7)	1 (8)	1 (7)		0 (0)	2 (9)		1 (11)	1 (6)	
7	3 (11)	1 (8)	2 (13)		1 (25)	2 (9)		0 (0)	3 (17)	

Additional analyses included the use of potential skin irritants like sunscreens, cosmetics, lotions, and topical medications. Still, none were found to have a significant association with *H. pylori* or SIBO positivity. For subjects with flushing, additional characteristics were analyzed for possible associations: frequency, duration, extent, severity, associated sweating, and associated hot flashes. Flushing triggers were also assessed: emotional stress, consumption of hot beverages and alcohol, spicy foods, exercise, cold or hot temperatures, and baths/showers. Ultimately, none of these flushing variables had significant associations with *H. pylori* colonization or SIBO. 

## Discussion

To contribute to the current understanding of rosacea pathogenesis, particularly as it relates to the gut microbiome, this study attempted to further describe the relationship between rosacea and *H. pylori* colonization and SIBO by controlling for several potential confounders that were lacking in similar prior studies, such as rosacea subtype, GI disease, previous GI surgery, medication use, immune suppression, autoimmune disease, and other comorbidities. Our study found that *H. pylori* colonization was not significantly higher in rosacea subjects compared to the general population, but we found a significantly higher proportion of SIBO when comparing to the mid-range population estimation of SIBO. Additionally, patient demographics and clinical characteristics were not associated with *H. pylori *colonization and/or SIBO.

Microorganisms remain an important topic of discussion for many medical conditions due to their recognized role in immune functioning. Cutaneous organisms like *Demodex folliculorum, Bacillus oleronius, Staphylococcus epidermidis*, and *Cutibacterium acnes* have been well studied in rosacea [17-19]. The overgrowth of these microorganisms is thought to contribute to rosacea pathogenesis through proposed mechanisms of aberrant Toll-like receptor 2 signaling, excessive cathelicidin expression, and reactive oxygen species, with downstream effects of unregulated inflammation, vasodilation, angiogenesis, and extracellular matrix deposition [7].

Just as microorganisms on the skin are associated with rosacea, contemporary studies have investigated a similar role of extracutaneous organisms in rosacea pathophysiology. In recognition that GI conditions represent the most frequent systemic comorbidities in rosacea, theories surrounding the gut-skin axis emerged. The conceptual model identifies a vital role for the gut microbiome in the regulation of skin immunity, whereby perturbations of the microbiota can compromise the integrity of the intestinal barrier, permitting the mobilization of bacteria and their metabolites. The systemic effects may disrupt skin homeostasis, resulting in cutaneous inflammatory manifestations like that of rosacea [20]. However, uncertainty remains as to whether abnormal microorganismal growth contributes to or results from such inflammation [18].

To further elucidate the relationship of gut dysbiosis with rosacea, several studies have investigated *H. pylori *and SIBO as representative extracutaneous microorganisms. Some studies have shown that *H. pylori* colonization is disproportionately higher in rosacea patients compared to population prevalence estimates or non-rosacea control groups [21-27], as high as 88% in rosacea patients compared to 65% in healthy controls in one study [27]. Beridze et al. further reported a significant association of *H. pylori* with rosacea severity [24]. However, not all studies have corroborated these findings [28-34]. Illustratively, Lazaridou et al. studied 100 rosacea subjects and 100 controls, finding no significant difference in *H. pylori* (42% and 46%, respectively) [35]. The disparate results may be due to differences in bacterial identification methods and inconsistent controlling for potential confounders. For *H. pylori*, a variety of testing methods were used in the aforementioned studies, including IgG/IgA/anti-CagA serological analyses, stool antigen tests, gastric biopsy, and ^13^C-UBT. Reports have since supported ^13^C-UBT as the best, non-invasive method of detection for* H. pylori*; therefore, we utilized this method of testing for our study [36]. 

Additionally, a lack of proper controlling was observed in many of the studies, where potential confounders such as rosacea subtype, medication use (antibiotics, NSAIDs, GI-modulating medications, rosacea treatments, etc.), GI disease, previous GI surgery (or colonic purging), immunosuppression, autoimmune disease, or comorbidities were not addressed in the inclusion/exclusion criteria and/or statistical analysis. The importance of such considerations was exemplified by Bonamigo et al., who found a significant difference in *H. pylori* proportions when adjusting for antibiotic use [37]. Another study considered GI symptoms and demonstrated a higher proportion of *H. pylori *in rosacea subjects with dyspeptic symptoms compared to those without dyspepsia [38].

A potential role for SIBO in rosacea was also proposed later in 2008 when Parodi et al. found that 46% of rosacea subjects (of 113) tested positive for SIBO compared to only 5% in the control group (of 60), with subsequent studies supporting a similar relationship [6,39]. However, as in *H. pylori*, other studies have demonstrated no difference in SIBO when comparing rosacea subjects with controls [40]. A systematic review demonstrated the limitations of SIBO detection methods as potentially contributing to the discrepant results, and a nationwide cohort study highlighted the challenges of SIBO prevalence estimation for use in comparative analyses [15,40]. Our study utilized glucose breath testing (for hydrogen and methane) as it provides the simplest and most widely available non-invasive diagnostic modality for SIBO diagnosis. However, this is not without its own shortcomings, as it only detects proximal SIBO because glucose is completely absorbed in the proximal jejunum, contributing to the lower sensitivity compared to other methods like lactulose breath testing and small bowel aspirate with culture. While lactulose may slightly improve sensitivity as a non-absorbable disaccharide, the specificity is lower than that of glucose, as the rise in breath hydrogen coincides with the arrival of lactulose in the cecum, raising false-positive rates. While small bowel aspirate is considered a “gold standard” test, it is invasive, and reports have since justified glucose breath testing as an acceptable means of study [12].

As noted above, past studies have demonstrated that confounding variables such as medication use, GI diseases, previous GI surgery, and others can profoundly affect *H. pylori *and SIBO testing results. Gravina et al., therefore, attempted to control several of these variables in their large study comparing 90 rosacea subjects to 90 controls [5]. They excluded subjects with a history of GI disease or surgery, pregnancy, taking proton pump inhibitors, histamine receptor antagonists, antibiotics, or NSAIDs (within two months), and other notable comorbidities. Ultimately, they found a significant difference in *H. pylori* between rosacea subjects and controls, but not SIBO. Our study emulated Gravina’s exclusion criteria with the added considerations of colonic purging or bowel preparation within two months, steroid use, immunosuppression, autoimmune disorders, renal or liver diseases, and isolated phymatous or ocular rosacea. Our study also considered subject demographics, rosacea characteristics, and GI symptoms.

Overall, despite the conflicting results of rosacea’s association with *H. pylori* and SIBO in the literature, there are notable similarities in the pathogenesis of rosacea and *H. pylori *and SIBO. Rosacea, an immune-mediated inflammatory disease, often presents with flushing. Likewise, *H. pylori* can induce systemic inflammation and facial flushing through the release of angiogenic and vasoactive factors. For instance, proposed increases in nitrous oxide from *H. pylori *may contribute to vasodilation, inflammation, and cytotoxic reactions. Additionally, inflammation may be propagated through an increased delivery of immunogenic proteins, cytotoxin-associated gene A, tumor necrosis factor (TNF)-α, and interleukin (IL)-8 [41]. Increases in gastrin levels may additionally contribute to observable flushing [42]. Additionally, SIBO is thought to produce toxic metabolites that may induce enterocyte injury. This injury to enterocyte integrity may result in increased intestinal permeability and unabated systemic transmission of inflammatory substances from the GI tract. The gut bacteria may also mimic immunogens, potentially leading to upregulation of TNF-α, suppression of IL-17, stimulation of T-helper 1 immunity, and subsequent development or exacerbation of rosacea [43-46].

Ultimately, the association of rosacea with *H. pylori* and SIBO remains without consensus in the current literature. We appreciate the need for additional, well-controlled studies. While this study is novel in its extensive exclusion criteria and consideration of various potential associations, we recognize the limitations in sample size and lack of a comparative control group. In power analysis, for 80% power to detect a difference in SIBO/*H. pylori* positivity among rosacea subtypes, we would have needed an enrollment sample of 115 participants. However, we achieved enrollment of 27 participants, limited by our extensive exclusion criteria, which was notably an important objective of this study, though practically restrictive to participant recruitment. Further, the small sample size and lack of a control group were subject to the confines of available funding resources. Furthermore, regarding the clinical applicability of our findings, the observational nature of the study design should be appreciated. While some other studies have further attempted eradication therapies, it remains unclear whether the antibiotic treatments of *H. pylori* or SIBO lead to the improvement of rosacea through means of eliminating the abnormal gut bacteria or rather due to systemic anti-inflammatory effects of the antibiotics themselves, leading to direct improvement in rosacea.

## Conclusions

While studies have reported a higher prevalence of *H. pylori* and SIBO in rosacea subjects, a significant discrepancy in the literature exists, likely attributed to inconsistent testing methods and incomplete controlling for potential confounders. In our study, extensive controlling of potential confounders like gastrointestinal disease, autoimmunity and immunodeficiency, comorbidities, medications, and rosacea characteristics revealed a significantly higher prevalence of SIBO, but not *H. pylori*, in rosacea subjects compared to their mid-range population estimations. These findings not only suggest an interplay between cutaneous pathology and systemic immunology, but also underscore the importance of considering disease pathophysiology in study design. We hope that this study effectively adds to the body of evidence exploring the potential role of GI bacterial overgrowth in rosacea pathogenesis. Ultimately, while SIBO may be associated with rosacea, however, it remains incompletely understood as to whether SIBO itself contributes to rosacea pathophysiology or if SIBO prevalence and rosacea concurrently are downstream effects of innate abnormalities in systemic immunity. Future studies are warranted to elucidate this relationship further. However, this observed association between rosacea and SIBO may provide a promising and novel therapeutic target in the treatment and management of individuals with rosacea.
